# Impact of grid size on uniform scanning and IMPT plans in XiO treatment planning system for brain cancer

**DOI:** 10.1120/jacmp.v16i5.5510

**Published:** 2015-09-08

**Authors:** Suresh Rana, Yuanshui Zheng

**Affiliations:** ^1^ Department of Medical Physics ProCure Proton Therapy Center Oklahoma City OK; ^2^ Department of Radiological Sciences University of Oklahoma Health Sciences Center Oklahoma City OK USA

**Keywords:** grid size, dose calculation, uniform scanning, IMPT, brain cancer

## Abstract

The main purposes of this study are to: 1) evaluate the accuracy of XiO treatment planning system (TPS) for different dose calculation grid size based on head phantom measurements in uniform scanning proton therapy (USPT); and 2) compare the dosimetric results for various dose calculation grid sizes based on real computed tomography (CT) dataset of pediatric brain cancer treatment plans generated by USPT and intensity‐modulated proton therapy (IMPT) techniques. For phantom study, we have utilized the anthropomorphic head proton phantom provided by Imaging and Radiation Oncology Core (IROC). The imaging, treatment planning, and beam delivery were carried out following the guidelines provided by the IROC. The USPT proton plan was generated in the XiO TPS, and dose calculations were performed for grid size ranged from 1 to 3 mm. The phantom containing thermoluminescent dosimeter (TLDs) and films was irradiated using uniform scanning proton beam. The irradiated TLDs were read by the IROC. The calculated doses from the XiO for different grid sizes were compared to the measured TLD doses provided by the IROC. Gamma evaluation was done by comparing calculated planar dose distribution of 3 mm grid size with measured planar dose distribution. Additionally, IMPT plan was generated based on the same CT dataset of the IROC phantom, and IMPT dose calculations were performed for grid size ranged from 1 to 3 mm. For comparative purpose, additional gamma analysis was done by comparing the planar dose distributions of standard grid size (3 mm) with that of other grid sizes (1, 1.5, 2, and 2.5 mm) for both the USPT and IMPT plans. For patient study, USPT plans of three pediatric brain cancer cases were selected. IMPT plans were generated for each of three pediatric cases. All patient treatment plans (USPT and IMPT) were generated in the XiO TPS for a total dose of 54 Gy (relative biological effectiveness [RBE]). Treatment plans (USPT and IMPT) of each case was recalculated for grid sizes of 1, 1.5, 2, and 2.5 mm; these dosimetric results were then compared with that of 3 mm grid size. Phantom study results: There was no distinct trend exhibiting the dependence of grid size on dose calculation accuracy when calculated point dose of different grid sizes were compared to the measured point (TLD) doses. On average, the calculated point dose was higher than the measured dose by 1.49% and 2.63% for the right and left TLDs, respectively. The gamma analysis showed very minimal differences among planar dose distributions of various grid sizes, with percentage of points meeting gamma index criteria 1% and 1 mm to be from 97.92% to 99.97%. The gamma evaluation using 2% and 2 mm criteria showed both the IMPT and USPT plans have 100% points meeting the criteria. Patient study results: In USPT, there was no very distinct relationship between the absolute difference in mean planning target volume (PTV) dose and grid size, whereas in IMPT, it was found that the decrease in grid size slightly increased the PTV maximum dose and decreased the PTV mean dose and PTV $D_{50\%}$. For the PTV doses, the average differences were up to 0.35 Gy (RBE) and 1.47 Gy (RBE) in the USPT and IMPT plans, respectively. Dependency on grid size was not very clear for the organs at risk (OARs), with average difference ranged from −0.61 Gy (RBE) to 0.53 Gy (RBE) in the USPT plans and from −0.83 Gy (RBE) to 1.39 Gy (RBE) in the IMPT plans. In conclusion, the difference in the calculated point dose between the smallest grid size (1 mm) and the largest grid size (3 mm) in phantom for USPT was typically less than 0.1%. Patient study results showed that the decrease in grid size slightly increased the PTV maximum dose in both the USPT and IMPT plans. However, no distinct trend was obtained between the absolute difference in dosimetric parameter and dose calculation grid size for the OARs. Grid size has a large effect on dose calculation efficiency, and use of 2 mm or less grid size can increase the dose calculation time significantly. It is recommended to use grid size either 2.5 or 3 mm for dose calculations of pediatric brain cancer plans generated by USPT and IMPT techniques in XiO TPS.

PACS numbers: 87.55.D‐, 87.55.ne, 87.55.dk

## I. INTRODUCTION

Interest in proton therapy for cancer treatment continues to grow in the medical community. Proton beams have finite range and sharp distal falloff.^(1)^ These physical properties of protons allow the deposition of majority of the radiation dose within the tumor volume while better sparing the critical structures. This could be advantageous for the patients seeking radiation treatment, especially to the pediatric patients. A recent publication by Moteabbed et al.^(2)^ investigated the risk of radiation‐induced secondary cancer and concluded that proton therapy is highly beneficial for pediatric patients when compared to photon therapy. Additionally, several studies have reported that dosimetric results of proton therapy are superior to that of photon therapy for the pediatric patients.^3^, ^4^, ^5^ It must be noted that dosimetric results in the treatment plans can be affected by the selection of beam delivery technique and treatment planning system (TPS). Furthermore, dose calculation grid size may have an effect on dose calculations as shown by studies on lung stereotactic body photon radiation therapy (SBRT)^(6)^ and on head and neck intensity‐modulated radiation therapy (IMRT).^(7)^ For treatment plans with high dose gradient in photon therapy, it has been recommended to use a smaller grid size, which can yield more accurate dose calculations, but for a longer computational time.^(8)^ Hence, treatment planning process may involve the compromise between the dose calculation accuracy and efficiency. The majority of previous studies on dosimetric impact of grid size have been done in photon therapy, and the effect of grid size on proton dose calculations for brain cancer is yet to be investigated, especially for the brain cancer treatment plans generated in the XiO TPS

In this technical report, we have investigated the dosimetric impact of dose calculation grid size on proton brain cancer plans, which typically include the regions of different tissue heterogeneity and dose gradient. The main purposes of this study are to: 1) evaluate the accuracy of XiO TPS for different dose calculation grid size based on head phantom measurements in uniform scanning proton therapy (USPT); and 2) compare the dosimetric results for various dose calculation grid sizes based on real computed tomography (CT) dataset of pediatric brain cancer patients in XiO TPS for the USPT and intensity‐modulated proton therapy (IMPT). To our best knowledge, this is the first study which investigates the effect of grid size in XiO TPS for pediatric brain cancer USPT and IMPT plans.

## II. MATERIALS AND METHODS

The USPT and IMPT plans were generated in XiO TPS (version 5.00., CMS Inc, Elekta, St. Louis, MO) with pencil beam algorithm.^(9)^ For the phantom study, proton beam delivery system^10^, ^11^ used for the measurements was the uniform scanning technology (IBA, Louvainla‐Neuve, Belgium). For both the phantom and patient studies, CT image sets were obtained for slice thickness of 1.25 mm.

### A. Phantom study

**Figure 1:**
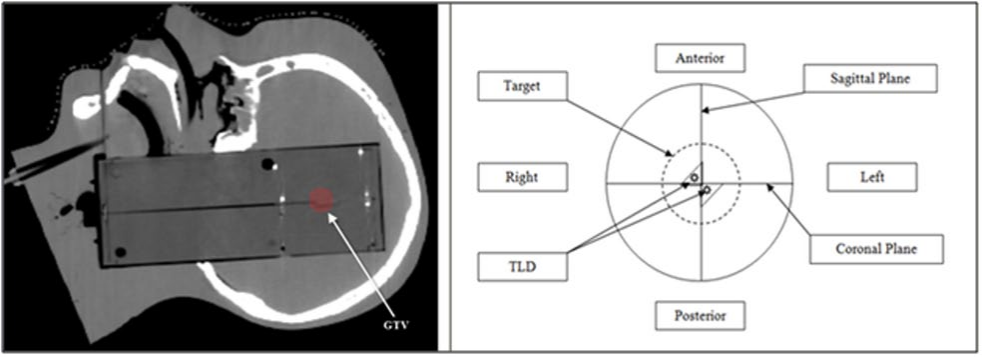
Sagittal view of CT image of the IROC brain phantom (left); cross‐sectional view of the IROC head proton phantom with the dosimetry insert (right).

In this study, we have utilized the anthropomorphic head proton phantom provided by Imaging and Radiation Oncology Core (IROC).^(12)^ The imaging, treatment planning, and beam delivery were carried out following the guidelines^(12)^ provided by the IROC. The CT image of the phantom with imaging insert was obtained (Fig. 1). The imaging insert was used to delineate the target structure (simulating a brain tumor).

The USPT plan was generated in the XiO TPS using three equally weighted fields (vertex, right lateral, and left superior oblique) with an objective of achieving: 1) total dose of 6 Gy (relative biological effectiveness [RBE]) to at least 95% of the gross target volume (GTV) (4.05 cc), 2) minimum dose of 5.1 Gy (RBE) to the GTV, and 3) maximum dose of 6.6 Gy (RBE) to 0.03 cc of the GTV. Dose calculations were performed using dose calculation grid size ranged from 1 to 3 mm, with an increment of 0.5 mm. For the uniform scanning proton beam delivery, the imaging insert was replaced by the dosimetric insert, which contained TLD capsules at two locations (right and left) near the center of the target, as well as two sheets of Gafchromic Dosimetry Media (International Specialty Products, Wayne, NJ) to provide dose distributions in the coronal and sagittal planes (Fig. 1). The phantom with the dosimetric insert was irradiated to approximately 6 Gy (RBE) using uniform scanning proton beams. The irradiated TLDs were read by the IROC. The calculated point doses from the XiO were compared to the measured TLD doses provided by the IROC. The IROC also provided the film analysis results (gamma index), which compared the measured dose distributions with the XiO‐calculated dose distributions (grid size calculation: 3 mm) in the coronal and sagittal planes for the USPT. Additionally, IMPT plan was generated using multiple‐field optimization (MFO) technique for a total dose of 6 Gy (RBE) based on the same CT dataset of the IROC phantom, and IMPT dose calculations were performed for grid size ranged from 1 to 3 mm. For a comparative purpose, calculated USPT and IMPT planar dose distributions at isocenter for different grid sizes (1, 1.5, 2, and 2.5 mm) were compared with that of 3 mm grid size using OmniPro‐I'mRT software, version 1.6.009 (IBA Dosimetry, Schwarzenbruck, Germany).

### B. Patient study

Three pediatric brain cancer cases treated with uniform scanning proton beams were selected for this study. First, the USPT plan for each case was generated for a total dose of 54 Gy (RBE) with dose per fraction of 1.8 Gy (RBE). Beam arrangement and weighting in each case were done with the objective of maximizing the target coverage and meeting the normal tissue dose constraints based on the institutional protocol. Second, for each pediatric brain cancer case, IMPT plan was generated using MFO technique for a total dose of 54 Gy (RBE) with dose per fraction of 1.8 Gy (RBE). The beam arrangements in the IMPT plans were kept the same as in the USPT plans. Current standard dose calculation grid size at our center is 3 mm. In order to investigate the dosimetric impact of grid size, treatment plans (USPT and IMPT) of each case was recalculated for grid sizes of 1, 1.5, 2, and 2.5 mm, and the results were compared with that of 3 mm grid size. Specifically, the absolute difference in various dosimetric parameters between the selected grid sizes (1, 1.5, 2, and 2.5 mm) and 3 mm grid size was calculated. The absolute difference (D) in each case is calculated as:

(1) D(g)=X(g)−X(3)

where X=dosimetric parameter (e.g., optic chiasm maximum dose, brain maximum dose); X(g)= dosimetric result of for a given grid size (g) (g=1, 1.5, 2, and 2.5 mm); and X(3)=dosimetric result of X for a standard grid size of 3 mm.

Additionally, dose computation time for each grid size was recorded and compared. The USPT and IMPT plans were calculated on HP Proliant DL380 G7 computer (RAM: 16 GB; CPU:2×Xeon X5650; Hewlett‐Packard, Palo Alto, CA).

## III. RESULTS

### A. Phantom study

Table 1 shows calculated doses of different grid sizes (1, 1.5, 2, 2.5, and 3 mm) compared to the measured TLD dose. The calculated doses varied slightly with grid size, with difference of ±0.1%. For the right TLD, on average, the calculated dose was higher than the measured dose by 1.49% (range, 1.42%–1.56%). For the left TLD, the calculated dose was higher than the measured dose by 2.63% (range, 2.55%–2.68%).

Table 2 shows the gamma analysis results comparing the calculated dose distributions of 3 mm grid size with the measured film distributions for uniform scanning proton beams. Based on the gamma index of IROC (5% and 3 mm), the passing rates were 100% and 99.65% in the coronal and sagittal planes, respectively. Table 3 shows the results of the gamma analysis between the standard grid size (3 mm) versus other grid sizes (1, 1.5, 2, and 2.5 mm) for the USPT and IMPT plans. For gamma index criteria 2% and 2 mm, the percentage of points meeting the criteria was found to be 100% in all three planes (axial, coronal, and sagittal) in the USPT and IMPT plans. For a stringent gamma index criteria 1% and 1 mm, the percentage of points meeting the criteria was from 98.61% to 99.97% for the USPT plans and from 97.92% to 99.85% for the IMPT plans.

**Table 1:** XiO TPS calculated doses for various grid sizes and measured TLD doses in two locations (right and left) near the center of the target for uniform scanning proton beams in brain phantom study

	*Measured*	*Dose (Gy [RBE]) Calculated values from XiO for different grid sizes*				
*Location*	*TLD*	*1 mm*	*1.5 mm*	*2.0 mm*	*2.5 mm*	*3.0 mm*
Right	604	612.6	612.7	613.2	613.4	613.0
Left	605	621.1	621.0	621.2	620.4	621.0

**Table 2:** Summary of gamma analysis comparing calculated dose distributions (3 mm grid size) in XiO TPS with measured film distributions for uniform scanning proton beams in brain phantom study

*Film Plane*	*Gamma Index* ^a^	*IROC Criteria*	*Result* ^b^
Coronal	100	≥85%	100.00%
Sagittal	100	≥85%	99.65%

a Percentage of points meeting gamma index criteria of 5% and 3 mm.

b Film analysis results provided by IROC.

**Table 3:** Gamma analysis comparison between standard grid size (3 mm) vs. other grid sizes (1, 1.5, 2, and 2.5 mm) for USPT and IMPT in brain phantom study. This analysis is based on the planar dose distributions obtained from the XiO TPS

	*Film Plane*	*3 mm vs. 2.5 mm*	*3 mm vs. 2 mm*	*3 mm vs. 1.5 mm*	*3 mm vs. 1 mm*
		*Percentage of Points Meeting Gamma Index Criteria of 1% and 1 mm*			
USPT	Axial	99.61%	98.90%	99.56%	99.97%
	Coronal	99.35%	98.91%	98.61%	98.64%
	Sagittal	99.18%	99.16%	99.03%	99.08%
IMPT	Axial	97.92%	99.85%	98.48%	98.46%
	Coronal	98.79%	99.13%	99.01%	99.09%
	Sagittal	98.31%	98.88%	98.29%	98.44%
		*Percentage of Points Meeting Gamma Index Criteria of 2% and 2 mm*			
USPT	Axial	100.00%	100.00%	100.00%	100.00%
	Coronal	100.00%	100.00%	100.00%	100.00%
	Sagittal	100.00%	100.00%	100.00%	100.00%
IMPT	Axial	100.00%	100.00%	100.00%	100.00%
	Coronal	100.00%	100.00%	100.00%	100.00%
	Sagittal	100.00%	100.00%	100.00%	100.00%

USPT = uniform scanning proton therapy; IMPT = intensity‐modulated proton therapy.

### B. Patient study

2, 3 show the absolute difference in various dosimetric parameters between the selected grid sizes (1, 1.5, 2, and 2.5 mm) and standard grid size (3 mm) in the USPT and IMPT plans, respectively. The results in 2, 3 are averaged over three clinical cases.

**Figure 2:**
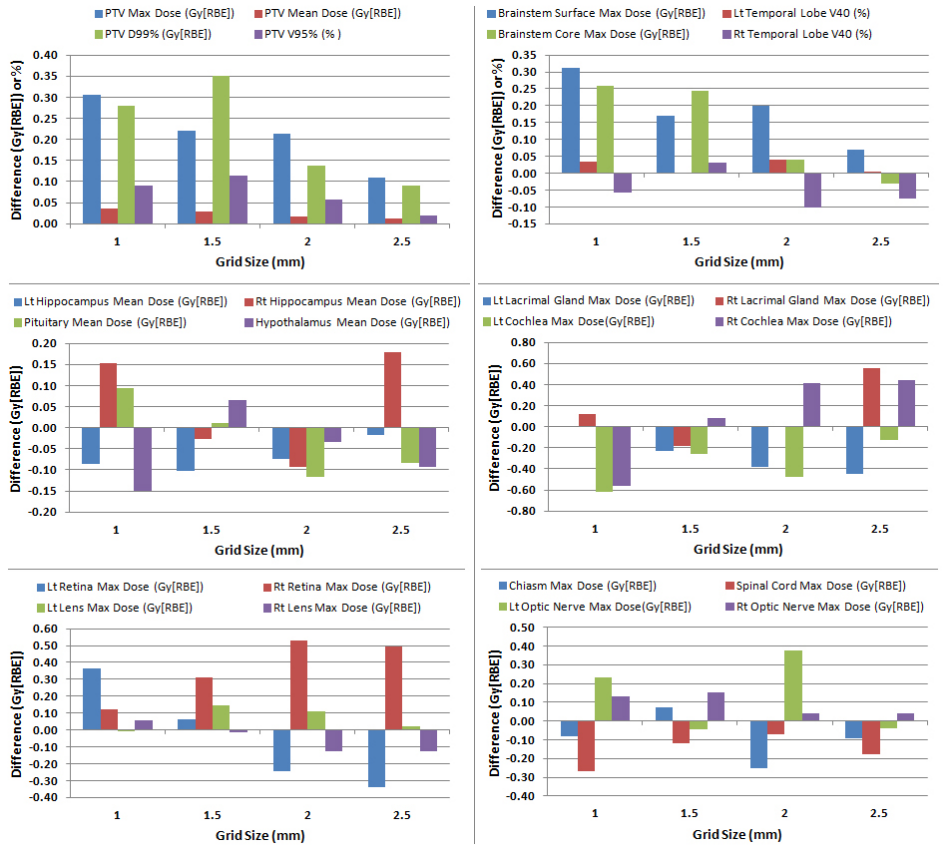
Absolute difference in dosimetric values between 3 mm grid size and selected grid sizes (1, 1.5, 2, and 2.5 mm) in uniform scanning proton therapy (USPT) brain cancer plans.

**Figure 3:**
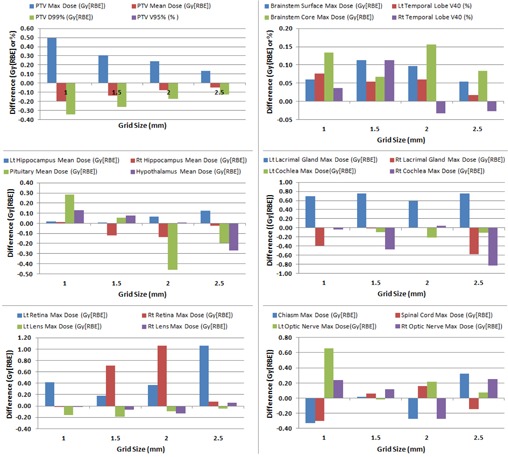
Absolute difference in dosimetric values between 3 mm grid size and selected grid sizes (1, 1.5, 2, and 2.5 mm) in intensity‐modulated proton therapy (IMPT) brain cancer plans.

#### B.1 PTV

As the grid size decreased from 2.5 mm to 1 mm, the difference in the PTV maximum dose slightly increased from 0.11 Gy (RBE) to 0.31 Gy (RBE) in the USPT plans and from 0.14 Gy (RBE) to 0.49 Gy (RBE) in the IMPT plans. For the PTV mean dose in the USPT plans, the grid sizes smaller than 3 mm produced slightly higher values when compared to 3 mm grid size, but the difference was very minimal (<0.05 Gy (RBE)). By contrast, the PTV mean dose in the IMPT plans slightly increased with an increase in grid size (absolute difference ranged from 0.05 Gy (RBE) to 0.5 Gy (RBE). No clear trend was obtained for the PTV coverage and $D_{99\%}$ in the USPT plans with absolute differences up to 0.11% for the target coverage and up to 0.35 Gy (RBE) for the $D_{99\%}$. The PTV coverage in the IMPT plans was 100% for all grid sizes. The $D_{99\%}$ in the IMPT plans slightly decreased with decrease in grid size with absolute differences ranged from 0.13 Gy (RBE) to 0.35 Gy (RBE).

#### B.2 OARs

The OARs results are also averaged over three clinical cases in 2, 3. There was no distinct relationship between the absolute difference in dosimetric parameter of OAR and dose calculation grid size for both the USPT and IMPT plans. The absolute differences in the mean dose to the hippocampus, pituitary, and hypothalamus were up to 0.18, 0.12, and 0.15 Gy (RBE), respectively, in the USPT plans and up to 0.14, 0.46, and 0.27 Gy (RBE), respectively, in the IMPT plans.

The differences in V40 of temporal lobe (left and right) were within ±0.11% for both the USPT and IMPT plans, whereas the absolute differences in the maximum dose to the lacrimal glands (left and right) were up to 0.65 Gy (RBE) in the USPT plans and up to 0.76 Gy (RBE). The absolute differences in the maximum dose to the cochlea (left and right) were up to 0.61 Gy (RBE) in the USPT plans and up to 0.83 Gy (RBE) in the IMPT plans. Similarly, the absolute differences in the maximum doses to retina were up to 0.53 Gy (RBE) and 1.06 Gy (RBE) in the USPT and IMPT plans, respectively, and the absolute differences in lens maximum dose were less than 0.20 Gy (RBE) in both the USPT and IMPT plans.

The absolute differences in the maximum dose to the cord were up to 0.27 Gy (RBE) and 0.30 Gy (RBE) in the USPT and IMPT plans, respectively, and the absolute differences in the maximum dose to the brainstem were up to 0.26 Gy (RBE) and 0.16 Gy (RBE) in the USPT and IMPT plans, respectively. For the optic chiasm, the absolute difference in the maximum dose was up to 0.25 Gy (RBE) in the USPT plans and up to 0.33 Gy (RBE) in the IMPT plans. For the maximum dose to the optic nerve (left and right), the absolute differences were up to 0.37 Gy (RBE) and 0.66 Gy (RBE) in the USPT and IMPT plans, respectively.

#### B.3 Dose computation time

**Figure 4:**
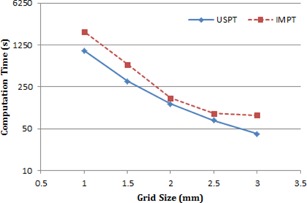
Dose computation time (seconds) for various grid sizes in XiO TPS for USPT and IMPT plans. The results are averaged over three pediatric brain cancer cases.

Figure 4 shows the dose computation time in seconds versus the grid size in XiO TPS for the USPT and IMPT plans. The results are averaged over three pediatric brain cancer cases. Dose computation time increased with the decrease in the grid size, and a sharp increase in the dose computation time was observed for grid size less than 2 mm for both the USPT and IMPT plans. As shown in Fig. 4, grid sizes of 3 and 2.5 mm resulted in relatively short calculation time, about 41 s and 69 s for USPT, and 84 s and 90 s for IMPT, respectively. The computational time of 2 mm grid size was about 129 s for USPT and 162 s for IMPT, and increased dramatically for smaller grid sizes (e.g., up to about 35 minutes for IMPT when 1 mm grid size was used). In general, the USPT plans had higher dose computation efficiency, with an average factor of 1.7 when compared to the IMPT plans.

## IV. DISCUSSION

Previous studies in photon therapy^6^, ^7^, ^8^ have recommended using smaller grid sizes for better dose calculation accuracy. Our phantom study results showed that the XiO calculated the dose within 3% of the measured TLD dose for all selected grid sizes in USPT. For both the right and left TLDs (Fig. 1), there was no distinct trend exhibiting the dependence of grid size on dose calculation accuracy when calculated dose of different grid sizes (1, 1.5, 2, 2.5, and 3 mm) were compared to the measured TLD doses (±3% uncertainty). Additionally, when the calculated dose of the largest grid size (3 mm) was compared to that of the smallest grid size (1 mm), the difference was found to be less than 0.1% in phantom study.

The gamma analysis was performed for both the USPT and IMPT plans generated based on the IROC phantom. Specifically, the planar dose distributions of test grid sizes (1, 1.5, 2, and 2.5) were compared against that of standard grid size (3 mm). The results showed very minimal differences among planar dose distributions of various grid sizes, with percentage of points meeting gamma index criteria 1% and 1 mm to be from 97.92% to 99.97%. The gamma evaluation using 2% and 2 mm criteria showed both the IMPT and USPT plans have 100% points meeting the criteria. This result suggests that grid size from 1 mm to 3 mm will not produce large dosimetric differences in the pediatric brain cancer treatment plans that are generated using USPT and IMPT techniques in the XiO TPS.

In patient study of three clinical cases, the decrease in grid size slightly increased the PTV maximum dose, thus resulting in an increase in the hotspot in the USPT and IMPT plans. However, the difference in PTV maximum dose between the 3 mm and 1 mm grid size plans was less than 0.5 Gy (RBE). Grid size did not have any major impact on the PTV coverage of USPT and IMPT plans. For the OARs, there was no clear relationship between the absolute difference in dosimetric parameter and dose calculation grid size, with average difference ranged from −0.61 Gy (RBE) to 0.53 Gy (RBE) in the USPT plans and from −0.83 Gy (RBE) to 1.39 Gy (RBE) in the IMPT plans.

**Figure 5:**
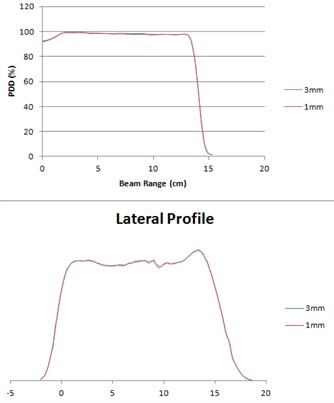
A comparison of PDDs/lateral profiles at 1 and 3 mm grid sizes for a lateral beam in case # 2.

The percent depth dose (PDD) and lateral profile within the target volume at various grid sizes produced very minimal or no differences. For example, Fig. 5 shows the PDD and lateral profile of right lateral uniform scanning proton beam within the PTV for the smallest grid size (1 mm) and the largest grid size (3 mm) from our patient study (case # 2). The PDD curves of both grid sizes (1 and 3 mm) were found to be almost identical in the distal region, and very minimal difference is seen in the SOBP and entrance regions. The penumbra regions of both grid sizes (1 and 3 mm) were also found to be similar to each other.

The results from our phantom and patient studies showed that there is minimal impact of grid size on proton dose calculations in USPT and IMPT planning of brain cancer when dose calculation grid size is varied from 1 mm to 3 mm in XiO TPS. The smaller grid size, however, can significantly increase the dose computation time in the XiO TPS, especially for a grid size smaller than 2.5 mm. Future studies need to address the impact of grid size in proton treatment planning (e.g., USPT and IMPT) for different commercially available TPS.

## V. CONCLUSIONS

The results based on our phantom study showed that the XiO TPS might slightly overestimate the dose in USPT, and the difference in the calculated dose between the smallest grid size (1 mm) and the largest grid size (3 mm) in phantom for USPT was typically less than 0.1%. Patient study results showed that the decrease in grid size slightly increased the PTV maximum dose in both the USPT and IMPT plans. However, no clear trend was obtained between the absolute difference in dosimetric parameter and dose calculation grid size for the OARs. Grid size has a large effect on dose calculation efficiency, and use of 2 mm or less grid size can increase the dose calculation time significantly. It is recommended to use grid size either 2.5 or 3 mm for dose calculations of pediatric brain cancer plans generated by USPT and IMPT techniques in XiO TPS.
